# Antibiotic Resistance Crisis: From Bacterial Bioprospecting to Artificial Intelligence

**DOI:** 10.1111/1758-2229.70267

**Published:** 2025-12-22

**Authors:** I. C. Cunha‐Ferreira, C. S. Vizzotto, J. Peixoto, R. H. Krüger

**Affiliations:** ^1^ Laboratory of Enzymology, Department of Cellular Biology University of Brasília (UnB) Brasília Brazil; ^2^ Molecular Biotechnology Centre Universidade de Brasília (UnB) Brasília Brazil; ^3^ Laboratory of Environmental Sanitation, Department of Civil and Environmental Engineering, Faculty of Technology University of Brasília (UnB) Brasília Brazil

**Keywords:** antimicrobial resistance, bioactive compounds, novel antibiotic development, therapeutic strategies

## Abstract

Antibiotics revolutionized medicine in the 20th century by drastically reducing mortality from bacterial infections. However, their effectiveness is threatened by the global rise of antimicrobial resistance (AMR), driven by misuse, overuse, and environmental dissemination. This review explores the historical trajectory of antibiotics, the mechanisms of bacterial resistance, and the urgent need for innovation amid a declining antibiotic development pipeline. Herein, we highlight the scientific and economic barriers that have discouraged investment by major pharmaceutical companies and examine emerging strategies to address this crisis. Key advances in microbial bioprospecting, including cultivation improvement techniques and genome mining, are discussed alongside the role of high‐throughput sequencing and bioinformatics in unlocking the metabolic potential of uncultivated microorganisms. Particular emphasis is placed on the integration of artificial intelligence and machine learning to accelerate drug discovery, predict antimicrobial activity, and identify resistance genes. Additionally, we present alternative therapeutic strategies beyond traditional antibiotics, such as phage therapy, antimicrobial peptides, quorum sensing inhibitors, synthetic conjugates, and vaccine development. Together, these interdisciplinary approaches offer promising pathways to revitalize the antimicrobial pipeline and address the growing threat of antibiotic resistance.

## Graphical abstract

Overview of the global antimicrobial resistance crisis and interdisciplinary approaches to address it, including bioprospecting, innovative cultivation strategies, genomics and bioinformatics, artificial intelligence and machine learning, alternative antimicrobial therapies, and educational strategies.

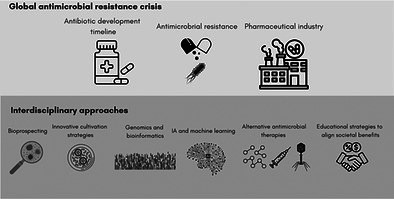

## 1 Introduction

The development of antibiotics has revolutionized health sciences and transformed modern society, significantly increasing lifespan and reducing mortality from infectious diseases. However, bacteria are highly adaptable organisms, capable of evolving in response to environmental pressures (MacGowan and Macnaughton 2017). When exposed to antibiotics that inhibit their growth, selective pressure favours genetic variants that confer resistance, which can then spread within and between bacterial populations (Munita and Arias 2016). These genetic variations arise spontaneously through mutations and disseminate naturally by vertical transmission or horizontal gene transfer—primarily through conjugation, and less frequently by transformation and transduction (Bello‐López et al. 2019). This evolutionary process drives the emergence of antibiotic resistance, enabling bacteria to withstand previously effective and available treatments (Lau et al. 2021).

**FIGURE 1:**
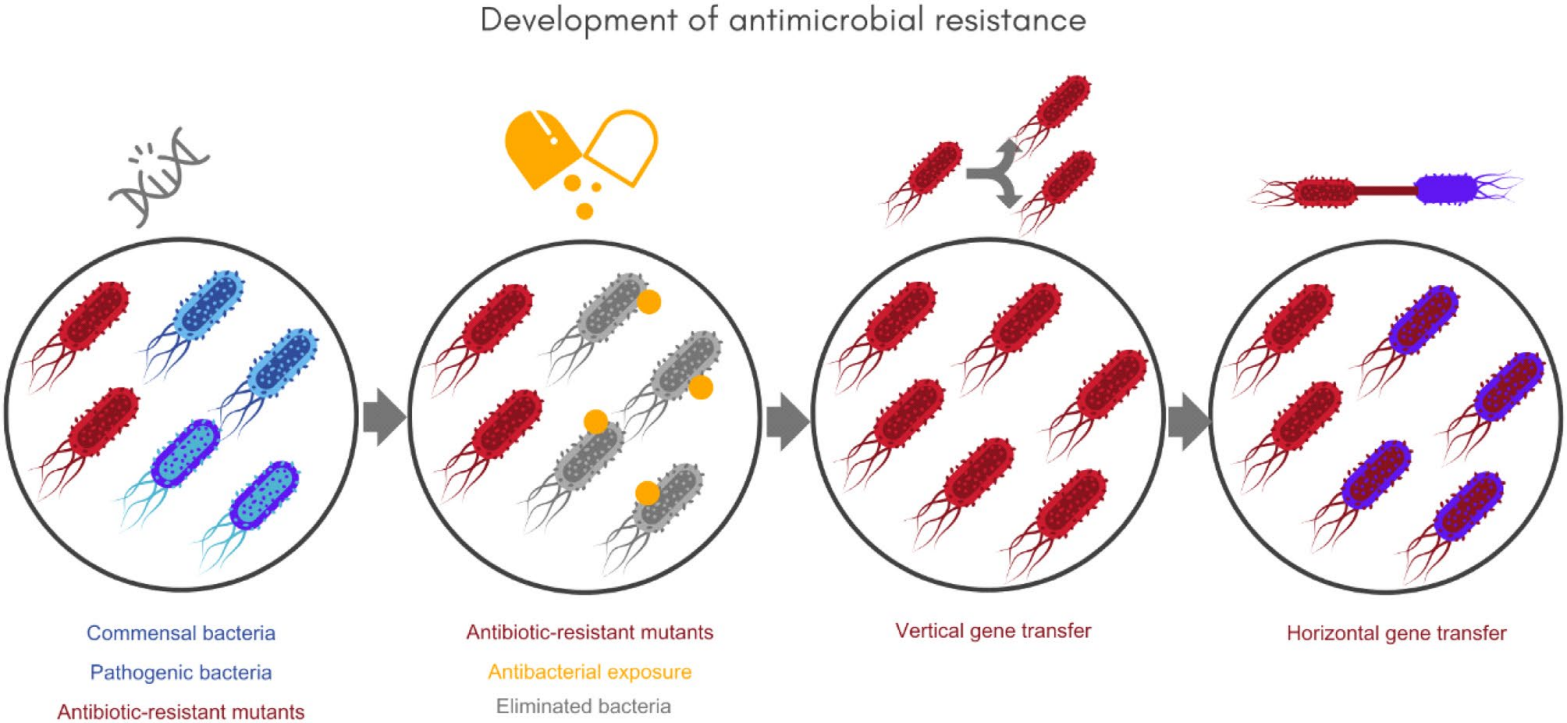
Genetic Mechanisms of Acquisition and Dissemination of Antimicrobial Resistance in Bacteria. Bacterial resistance may emerge through (i) spontaneous mutations during DNA replication, which are selected in the presence of antibiotics and transmitted vertically, or (ii) horizontal gene transfer via mobile genetic elements such as plasmids, transposons, and integrons. These mechanisms contribute to the rapid spread of multidrug resistance.

Antibiotic resistance, a consequence of bacterial evolution through natural selection, has emerged as a major threat to the effective treatment of common infections (Baquero et al. 2021) (Figure 1). This challenge is potencialized by the widespread overuse and misuse of antibiotics, including inaccurate diagnoses, inappropriate prescribing practices and self‐medication (Chokshi et al. 2019). An additional concern lies in the extensive use of antimicrobial agents in livestock, which contributes to the dissemination of resistant bacteria. Human exposure can occur through the consumption of contaminated animal products, as well as direct and indirect contact with animals, representing significant mechanisms for the spread of resistance (Toutain et al. 2016). In addition, there is the stagnation in the development of new antibiotics, particularly within the private sector, where economic disincentives and low commercial returns have led to a declining investment in antibiotic innovation (Brown and Wright 2016; PEW Funds 2019).

**FIGURE 2:**
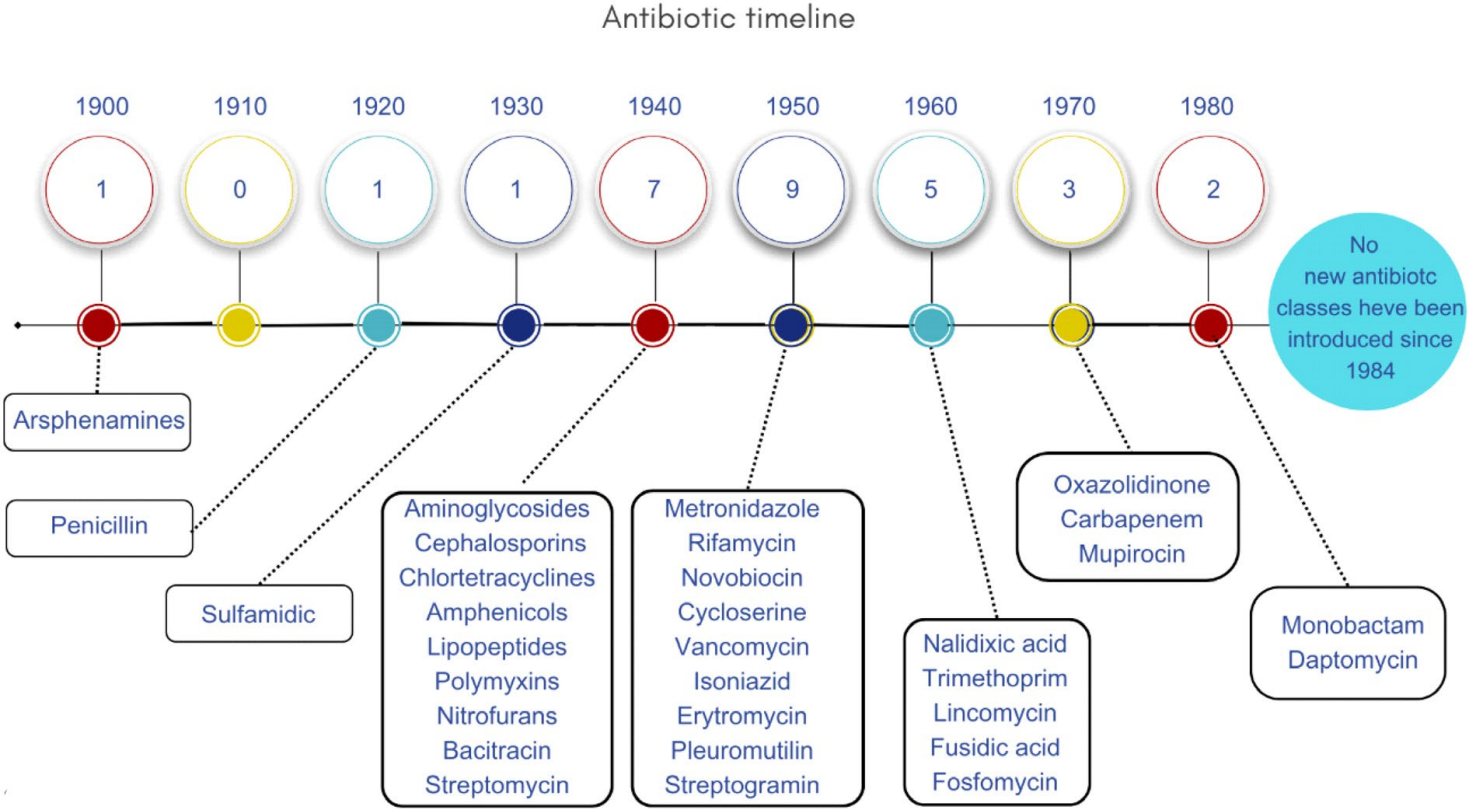
Timeline of antibiotic class discovery and decline in innovation. This figure illustrates the chronology of antibiotic class discovery. Milestones include arsphenamine (1909), penicillin (1928), and the “golden age” of discovery during the 1950s. The most recent new class of antibiotics was introduced in 1987, highlighting the current stagnation in antibiotic innovation (Silver 2011).

Historically, the emergence of antibiotic resistance was mitigated by the continuous discovery and clinical introduction of new antibiotics (Figure 2). As resistance arose, the pharmaceutical industry responded by developing a steady pipeline of novel compounds or improved derivatives capable of overcoming existing resistance mechanisms (Cook and Wright 2022). However, in recent decades, most major pharmaceutical companies have withdrawn from antibiotic research and development due to financial and regulatory challenges related to AMR. The burden of innovation has since shifted to smaller enterprizes, including start‐ups and biotechnology companies (Nicolaou and Rigol 2018; Plackett 2020; Uddin et al. 2021).

Meanwhile, an overreliance on high‐throughput screening and combinatorial chemistry has frequently led to the discovery of highly lipophilic molecules with poor aqueous solubility, resulting in suboptimal pharmacokinetic profiles (Lipinski et al. 2001). These scientific and economic barriers have driven up the cost of antibiotic discovery and development. Thus, the number of companies with financial capacity and willingness to absorb these risks has gradually declined, accelerated by merges and acquisitions over the past years (Paul et al. 2010; Kiriiri et al. 2020). In response, several initiatives have emerged to reinvigorate the antibiotic pipeline, including push and pull incentives, public‐private partnerships, and the involvement of non‐profit organisations aimed at de‐risking investment and supporting early‐stage research. Yet, sustained market‐driven strategies are needed to encourage and support the development of new antibiotics, alongside more eficiente discovery pipelines capable of replacing ineffective drugs and targeting resistant microorganisms (Uddin et al. 2021).

**FIGURE 3:**
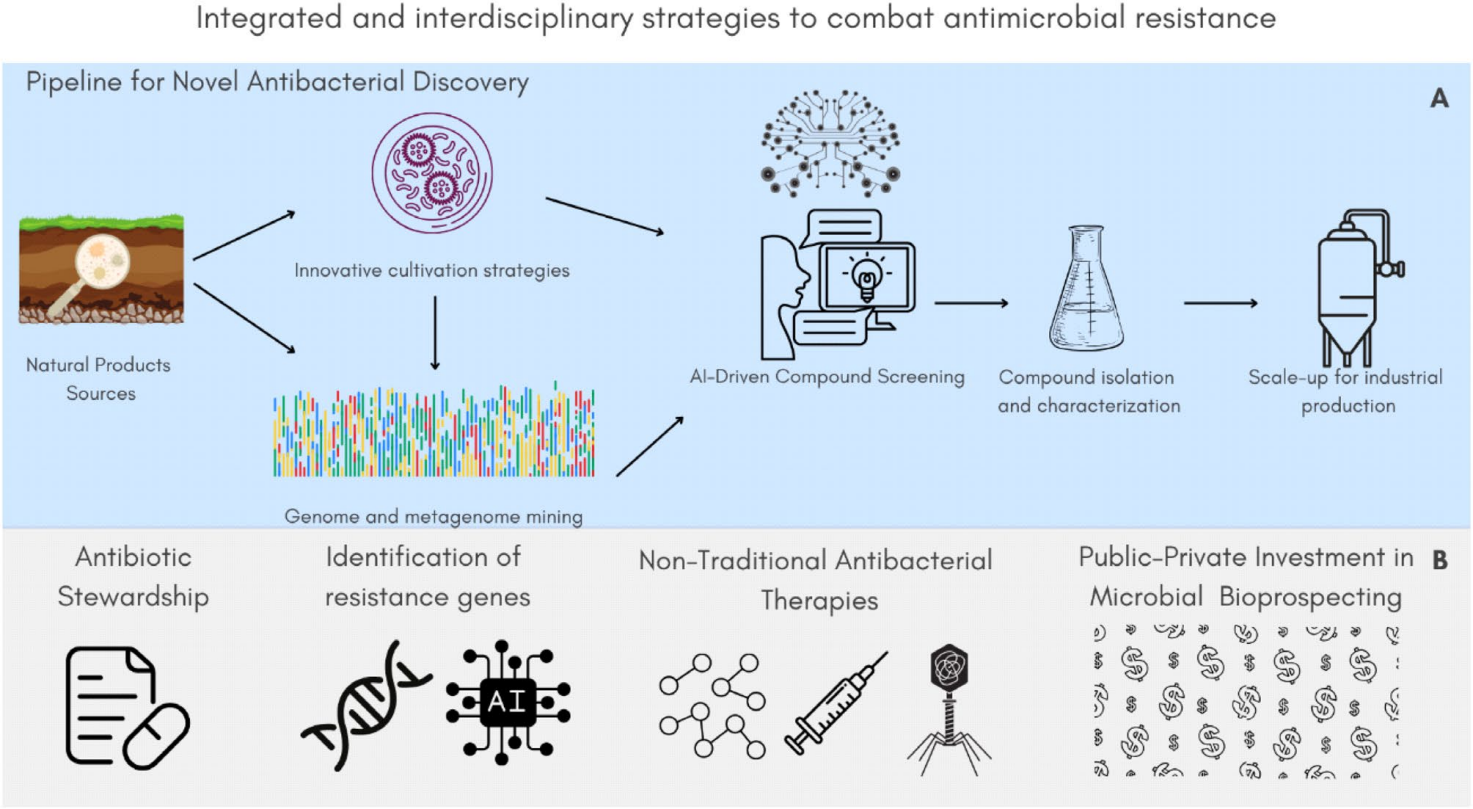
Integrated and interdisciplinary strategies to combat antimicrobial resistance. A multidimensional response to antimicrobial resistance must integrate: (A) innovation in microbial bioprospecting and drug discovery using modern tools such as genome mining and AI; and (B) coordinated efforts in antibiotic stewardship, investment in alternative therapies (e.g., phage therapy, AMPs), and robust financial and policy support for research and development.

**FIGURE 4:**
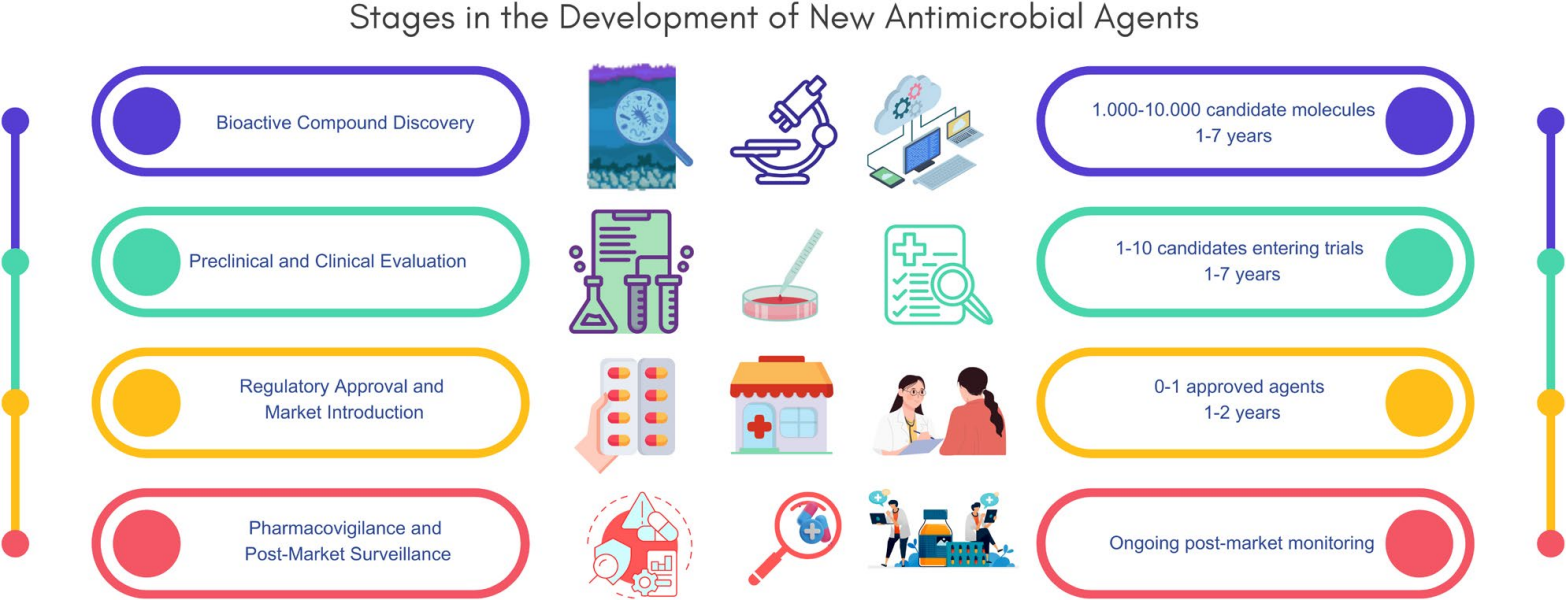
Stages in New Antimicrobial Development. Timeline of new antimicrobial development stages. The discovery, development, and regulatory approval of new antimicrobial agents can take between 8 and 18 years. This figure outlines the stages from microbial bioprospecting through preclinical research, clinical trials, and final market authorization, emphasizing the complexity and duration of the process (Miethke et al. 2021).

Microorganisms themselves may hold the key to addressing this demand, particularly when coupled with modern technologies such as high‐throughput genetic sequencing and artificial intelligence (AI), which can optimize bioprospecting efforts (Figures 3A and 4). Significant progress has been made through the study of microorganisms—from early descriptive analyses of pathogens to comprehensive characterisation of microbiomes across diverse environments, largely enabled by advances in sequencing technologies (Newman and Cragg 2020). These tools have facilitated not only the discovery of novel microbial species with antibiotic‐producing potential (Ling et al. 2015), but also the identification of co‐occurring antibiotic resistance genes across various ecological niches (Li et al. 2015; Kapinusova et al. 2023). Despite this progress, microbial diversity remains vastly untapped, whereas taxonomic markers analyses continue to reveal the presence of previously undescribed bacterial taxa, highlighting the immense biotechnological potential harboured in natural ecosystems (Gutleben et al. 2020).

Furthermore, genomic data suggests that even among known microorganisms, the full repertoire of bioactive compounds remains unexplored (Maithani et al. 2022). This has led to renewed interest in microbial natural products such as antimicrobial peptides (AMPs), including both ribosomally or non‐ribosomally synthesized compounds. Among these, bacteriocins consist in promising candidates to address antibiotic resistance. They have demonstrated a wide applicability, from extending the shelf life of food products to controlling human and veterinary pathogens, as well as modulating microbial populations in fermentation systems (Grady et al. 2016).

The discovery, isolation, and development of new antimicrobials is a lengthy and complex process, often requiring years of preclinical research and clinical trials to ensure safety and efficacy (Figures 3A and 4). Despite these efforts, many candidate compounds ultimately fail to demonstrate sufficient antimicrobial activity in vivo (Rondón‐Villarreal et al. 2014; Hughes and Karlén 2014; Lau et al. 2021). To minimize this risk, *in silico* machine learning approaches have been increasingly used as preliminary screening tools, enabling the identification of proteins with potential antimicrobial activity prior to laboratory validation (Lee et al. 2017) (Figures 3A and 4). AI also plays a key role in addressing AMR by facilitating the rapid and accurate identification of resistant bacterial strains (Figure 3B). This not only supports the development of novel therapeutic strategies but also helps reduce inappropriate antibiotic use through more accurate prescriptions (Lau et al. 2021). Together, these insights highlight the challenges and opportunities behind this urgent need for innovation in the antibiotic discovery and development pipeline. This review emphasizes the potential of prokaryotes as a valuable resource, and remarks the relevance of advances in microbiology, molecular biology, genetics, bioinformatics, and AI as complementary tools to overcome this growing global health crisis (Figure 3A,B).

## 2 Importance and History of Antibiotics

Antibiotics represent one of the most transformative class of pharmaceuticals and rank among the most impactful innovations of the 20th century. Their introduction marked a turning point in the fight against bacterial infections, saving millions of lives and reshaping the course of modern medicine (Cunha et al. 2019). Prior to their widespread use, infectious diseases accounted for over half of all global deaths (Armstrong et al. 1999). With the advent of antibiotics and the implementation of improved infection control measures, mortality from bacterial infections declined dramatically. As a result, global life expectancy increased by one to two decades over the spam of less than a century.

Chemical compounds were the first antibacterial agents discovered. In 1909, Paul Ehrlich created arsphenamine, an arsenic analog that was efficient against *Treponema pallidum* , the causal cause of syphilis (Haider et al. 2023). The first antibiotic to be discovered was penicillin, identified accidentally by Alexander Fleming in 1928 (Figure 2). He observed that a mould contaminating a Staphylococcus culture inhibited bacterial growth in its vicinity. The mould was later identified as a fungus of the genus Penicillium, initially classified as *Penicillium notatum* and subsequently reclassified as *Penicillium chrysogenum*. The substance it produced—penicillin—exhibited strong bactericidal activity and gave rise to the antibiotic era. Since then, thousands of antimicrobial compounds have been identified, described and brought to market (Tan and Tatsumura 2015) (Figure 2). In the 1940s, antibiotics were defined as substances produced by microorganisms that inhibit the growth of, or kill, other microbes (Cunha et al. 2019). Many of the antibiotics still in use today are derived from natural microbial products (Durand et al. 2019).

The 1950s marked the “golden age” of antibiotic discovery, during which major classes such as tetracyclines, cephalosporins, aminoglycosides, and macrolides were introduced (Nicolaou and Rigol 2018) (Figure 2). In subsequent decades, the genus *Streptomyces* became specially important as a source of novel antimicrobials, including actinomycin (derived from *Streptomyces* spp.), neomycin (derived from *S. fradiae* ), and streptomycin (derived from *S. griseus* ), many of which remain in clinical use (Cunha et al. 2019) (Figure 2).

These compounds act through diverse mechanisms, such as inhibition of cell wall synthesis, protein synthesis, nucleic acid synthesis and metabolic pathways, as well as membrane disruption (Table 1). Antibiotics are also subclassified based on their effects—bactericidal agents kill bacteria, whereas bacteriostatic agents inhibit their growth reversibly until the compound is degraded or removed (Kapoor et al. 2017). However, despite their historical success, antibiotics are increasingly compromized by bacterial resistance, which diminishes their long‐term efficacy (La Rosa et al. 2022).

**TABLE 1:** Mechanisms of action of major antibiotics classes.

Mode of action	Target	Drug class	Drugs examples
Disruption of cell wall biosynthesis	Penicillin‐binding protein (PBPs)	Beta‐lactams	Penicillin G, amoxicillin, and cephalosporin C
Inhibition of protein synthesis	Peptidoglycan subunits 30S ribosomal subunit	Glycopeptides, Aminoglycosides and tetracyclines	Vancomycin, Streptomycin, gentamicin, neomycin, tetracycline, and doxycycline
Inhibition of protein synthesis	Peptidoglycan subunits 50S ribosomal subunit	Macrolides, chloramphenicol, and oxazolidinones	Erythromycin, azithromycin, chloramphenicol, and linezolid
Inhibition of nucleic acid synthesis	Ribonucleic acid (RNA) ‐ RNA Polymerase	Rifamycin	Rifampin
Inhibition of nucleic acid synthesis	Deoxyribonucleic acid (DNA) ‐ Topoisomerases	Fluoroquinolones	Ciprofloxacin and ofloxacin
Antimetabolite ‐ activity	Folic acid synthesis	Sulfonamides and trimethoprim	Sulfamethoxazole, dapsone, and trimethoprim
Disrupt membrane integrity	Lipopolysaccharides (LPS)	Polymyxins	Polymyxin B and colistin

*Note:* This table represents the classification of antimicrobial agents according to their mode of action, primary biological targets, corresponding drug classes, and representative compounds.

*Source:* Uddin et al. (2021).

## 3 Antimicrobial Resistance

In 2017, the World Health Organization classified AMR as one of the most urgent threats to global public health, remarking the critical need for the development of new antibiotics (Tillotson and Zinner 2017). Among the most concerning pathogens are those grouped under the acronym ESKAPE, which includes *Enterococcus faecium, Staphylococcus aureus, Klebsiella pneumoniae*, *Acinetobacter baumannii, Pseudomonas aeruginosa* and *Enterobacter* spp.—bacteria that are not only highly virulent but also exhibit multidrug resistance. In parallel, the rise of multidrug‐resistant *Mycobacterium tuberculosis* further amplifies the global AMR crisis, particularly given its persistent and transmissible nature (Vajs et al. 2017). Thus, the growing prevalence of antibiotic resistance has driven initiatives to improve antimicrobial stewardship (AMS), including efforts to restrict inappropriate prescriptions in clinical and community settings. It has also led to the implementation of policies in many countries aimed at reducing antibiotic use in food‐producing animals (Sánchez et al. 2021; Kasimanickam et al. 2021).

AMR remains a critical barrier to the continued efficacy of antibiotics. While many pathogens are still susceptible to standard treatments, some exhibit intrinsic resistance due to structural or functional features, such as the outer membrane of Gram‐negative bacteria and the production of β‐lactamases. However, the biggest concern lies in the acquisition of resistance, which has become increasingly prevalent due to evolutionary pressures in clinical and environmental settings. Resistance arises primarily through two mechanisms: (i) horizontal gene transfer, which allows bacteria to acquire resistance genes from other organisms via plasmids, transposons, or integrons; and (ii) spontaneous mutations during DNA replication that generates resistant variants, which are then selected for in the presence of antibiotics and propagated vertically through bacterial populations (Figure 1). These mutations can alter drug targets, reduce membrane permeability, or lead to the overexpression of efflux pumps—mechanisms that enable bacteria to survive antibiotic exposure and proliferate (Table 1). Among these, mobile genetic elements (e.g., plasmids and transposons) constitute a particular challenge, as they frequently carry multiple resistance genes and can rapidly disseminate across bacterial species (Figure 1). Their presence not only confers multi‐drug resistance but also accelerates the spread of resistance in both clinical and agricultural environments (Cook and Wright 2022).

Although bacterial resistance was observed even during the early years of antibiotic use, the continuous discovery of new antibiotic candidates provided alternative treatment options (Dodds et al. 2020). However, by the 1980s, he pace of discovery began to decline sharply (Figure 2). The last novel class of antibiotics to reach the market was introduced in 1987, and the last major group of broad‐spectrum agents, the fluoroquinolones, was also developed during that decade (Durand et al. 2019) (Figure 2). Since then, antibiotic innovation has slowed considerably, and few new classes currently in development show potential against multidrug‐resistant infections (Frieri et al. 2017) (Figure 2).

## 4 Crisis in the Pharmaceutical Industry

After several productive decades of antibiotics discovery from environmental microorganisms—the primary source of known antimicrobial agents—the pharmaceutical industry experienced a significant decline in innovation starting in the 1980s (Figure 2). In response, companies increasingly adopted target‐based strategies enabled by advances in genomics and protein expression technologies, shifting from traditional natural product screening toward synthetic chemistry approaches (Årdal et al. 2020). Synthetic chemistry had previously led to important drugs, such as isoniazid, pyrazinamide, and ethambutol. Other synthetic agents, including metronidazole and oxazolidinone, and nalidixic acid (i.e., precursor of quinolones) were also added to the antimicrobial arsenal (Table 1). This approach was key to expand the utility of existing antibiotics, leading to analogues with broader spectra of activity against resistant pathogens (e.g., penicillin to ampicillin, and erythromycin to azithromycin) (Lewis 2020). Nevertheless, resistance eventually emerged even against these modified agents (Brüssow 2024).

The availability of whole‐genome sequencing (WGS) in the mid‐1990s reignited interest in antibiotic discovery by allowing target‐based screening of bacterial genomes. Yet, despite initial optimism, these efforts yielded few successful drug candidates relative to the substantial time (10–15 years) and cost invested, especially when compared with drug discovery outcomes in other therapeutic areas (Brüssow 2024).

Several factors have contributed to the declining attractiveness of the antibiotic market for developers: (i) antibiotics are generally less profitable than other drug classes due to national stewardship programs that restrict their use, their declining efficacy caused by AMR, and their short treatment durations and relatively low pricing, which limit commercial returns (Power 2006); (ii) regulatory pathways for antibiotic approval in the United States and the European Union remain complex, unpredictable, and subject to change, creating additional barriers and risks for developers (Projan 2003); and (iii) pharmaceutical companies have increasingly diverted scientific expertize and investment toward more lucrative therapeutic areas, as repeated market failures have rendered antibiotic development economically unviable. The high societal value of addressing AMR is not adequately captured by current market mechanisms (Butler et al. 2013). As a result, low return on investment, prolonged development timelines, and global recommendations to reserve novel antibiotics for last‐resort use have prompted many major pharmaceutical companies to exit the antibiotic R&D space altogether (Renwick et al. 2016; Årdal et al. 2020).

The decline in investment in antibiotic development also reflects the scientific challenges involved in candidate identification and early‐stage drug discovery (Anderson et al. 2023). While venture capital funding for cancer therapeutics has steadily grown, reaching $7 billion in 2020, investment in antibiotic research has remained stagnant, accounting for less than $250 million per year over the same period (McKenna 2024). Once led by the pharmaceutical and biotechnology sectors, antibiotic discovery is now increasingly reliant on academic research, as the number of industry players in this field has substantially dropped over the past two decades. Given the high failure rate associated with translating early discoveries into clinically viable therapies, there is a growing need to prioritize policies and funding mechanisms that foster innovation and support a robust antibiotic development pipeline (Cook and Wright 2022). Without renewed investment and strategic coordination, we risk entering a post‐antibiotic era, a future in which infections once routinely cured by mid‐20th‐century may again become untreatable (Cook and Wright 2022).

## 5 Microorganisms and Bioprospecting

The development of new antimicrobial agents from environmental microorganisms involves a multi‐step process, which begin with the selection, identification, and characterisation of microbial strains capable of producing bioactive metabolites (Figure 3A). Given the economic and scientific potential of bacterial‐derived bioproducts, bioprospecting are crucial to uncover novel compounds and their corresponding producer microbe. This knowledge supports subsequent stages in the drug discovery pipeline, including compound isolation and characterisation, optimization of culture conditions for metabolite production, scale‐up for industrial manufacturing, and, ultimately, preclinical and clinical trials (Amin et al. 2020) (Figures 3A and 4).

To survive in competitive environments, many microorganisms produce small molecules known as secondary metabolites or natural products, which often act as growth inhibitors of competing organisms. This ecological function makes them a valuable source of antibacterial compounds (Caballero‐Flores et al. 2023). Among the most promising microbial sources are those inhabiting extreme environments. Extremophilic and extremotolerant microorganisms, adapted to conditions at the limits of life, are known to produce structurally unique enzymes and metabolites with broad biotechnological applications (Quinn and Dyson 2024).

However, bioprospecting is not limited to extreme environments. *Actinobacteria*, particularly members of the genus *Streptomyces*, are prolific producers of bioactive natural compounds and are widely distributed in both terrestrial and aquatic environments (van Bergeijk et al. 2020). *Actinomycetes* (Phylum *Actinobacteria*) are especially valued for their capacity to synthesize a diverse array of essential metabolites, including compounds with activity against both prokaryotic and eukaryotic organisms (Takahashi 2017; Srivastava et al. 2019).

Many environmental bacteria are capable of producing between 20 and 40 distinct natural products—while filamentous fungi may produce even more—and a significant portion of these metabolites are antibiotics. Due to their metabolic flexibility and ecological adaptability, microorganisms constitute ideal models for biotechnological innovation. Numerous complex bioprocesses rely on the unique capability of microbial systems. Indeed, the majority of known antibiotics are derived from prokaryotes that evolved to produce these compounds for competition or communication within complex microbial communities (Durand et al. 2019).

One of the major limitations in studying microbial diversity and discovering new bioactive compounds is the inability to culture the vast majority of microbes found in natural ecosystems (Overmann et al. 2017). This limitation was first recognized in the mid‐1980s, when researchers observed a striking discrepancy between the number of bacterial cells visible under the microscopic and the number capable of forming colonies on standard culture media. This phenomenon, later termed the “great plate count anomaly”, highlighted the gap between microbial presence and cultivability (Staley and Konopka 1985). To overcome the limitations of traditional cultivation, modern approaches such as metagenomics and single‐cell sequencing have expanded access to the genetic and biosynthetic potential of uncultivable microorganisms. These advances have significantly broadened the scope of bioprospecting and natural product discovery.

## 6 Cultivation Improvement Techniques

Traditional cultivation methods have largely relied on empirical approaches, testing different media compositions and incubation conditions, then monitoring the growth and subculturing the isolates of interest. While effective for microorganisms whose physiological requirements are well understood, this strategy is limited in scope and often fails to capture the vast diversity of uncultured microbial taxa. To advance microbial ecology, biotechnology and public health, it is essential to develop targeted cultivation techniques capable of isolationg previously undescribed and yet‐to‐be‐cultivated microorganisms of biotechnological relevance (Overmann et al. 2017).

For many years, microorganisms were perceived as self‐sufficient entities with simple replication cycles, incapable of intercellular communication or social organization (Njoroge and Sperandio 2009). Yet, it is now well established that many prokaryotes engage in *quorum sensing*, a chemical communication system that enables population‐wide coordination through the production, release, detection, and response to small signalling molecules known as autoinducers. These hormone‐like molecules allow bacteria to assess the cell density and regulate collective behaviours such as light production, biofilm formation, virulence factor expression, cell aggregation, and genetic competence (Gray et al. 2013). In Gram‐negative bacteria, this is often mediated by acyl‐homoserine lactones (AHLs), whereas Gram‐positive species rely on short peptides. Other metabolic interactions involve molecules such as siderophores, which play dual roles in nutrient acquisition and microbial signalling (Papenfort and Bassler 2016; Soares 2022).

Traditional culture techniques, which often involve growing bacteria in isolation under nutrient‐rich conditions, fail to replicate the complex ecological interactions found in natural environments (Ho et al. 2017). For instance, soil microbiomes harbour highly diverse microbial communities that participate in dynamic biochemical exchanges and biogeochemical cycling. In these systems, microbial associations frequently share metabolites and growth‐promoting compounds, including quorum sensing signals that are essential for the survival of many uncultured taxa (Schink 2002; Jacoby et al. 2017; Bahram et al. 2018). In contrast, the absence of neighbouring species and their metabolites in pure culture result in a poor microbial recovery (Pande and Kost 2017). Therefore, most microorganisms resist cultivation under laboratory conditions, representing a major limitation in accessing the full breadth of biochemical diversity.

To overcome these challenges, it is essential to both refine existing cultivation techniques and develop innovative methods that better simulate the microorganisms' natural habitats (Kapinusova et al. 2023). Improvements may include the use of selective or restrictive media, antimicrobial gradients, or enrichment strategies that enhance the recovery of specific microbial groups (Bachmann et al. 2014; Overmann et al. 2017). Aditional approaches include co‐culturing target microorganisms with growth‐promoting partners, either through direct contact or via the use of helper strain supernatants (Stewart 2012; Boilattabi et al. 2021; Cunha‐Ferreira et al. 2024).

Innovative systems such as diffusion chambers, membrane‐based cultivation platforms, and bioreactors have been designed to facilitate microbial growth by allowing chemical exchange with the environment while maintaining physical separation (van Dorst et al. 2016; Pascual et al. 2014; Chaudhary et al. 2019; Boilattabi et al. 2021). Among these, in situ cultivation—the growth of microorganisms directly within their native environments—has shown promise in improving recovery of previously uncultivated species (Bollmann et al. 2007; Remenár et al. 2015).

Several devices have been developed to enable this strategy (Pascual et al. 2014). Kaeberlein et al. (2002) introduced a diffusion chamber in which seawater solidified with agar was enclosed between two polycarbonate membranes. This setup allowed the diffusion of nutrients from the surrounding environment into the inoculated agar while physically isolating the microbes (Kaeberlein et al. 2002). Similarly, the soil substrate membrane system developed by Ferrari et al. (2005) enabled microbial growth on a polycarbonate membrane that allowed nutrient flow from natural substrates.

For liquid‐based in situ cultivation, Aoi et al. (2009) designed the hollow fibre membrane chamber, consisting of porous polyvinylidene tubes that allows the diffusion of environmental molecules into the interior chamber where microorganisms are inoculated. Another example includes a liquid diffusion bioreactor, separated from the surrounding environment by a semi‐permeable polycarbonate membrane (Chaudhary et al. 2019; Chaudhary and Kim 2019).

Accordingly, isolating and maintaining novel microbial strains with potential antimicrobial activity requires significant experimental efforts. Still, these efforts can be minimized through predictive insights into microbial physiology and metabolism, enabled by ecological, genomic, metagenomic and physiological data obtained by culture‐independent methods, particularly through advances in genetic sequencing (Overmann et al. 2017).

## 7 Sequencing and Bioinformatics Tools for Microbial Bioprospecting

**FIGURE 5:**
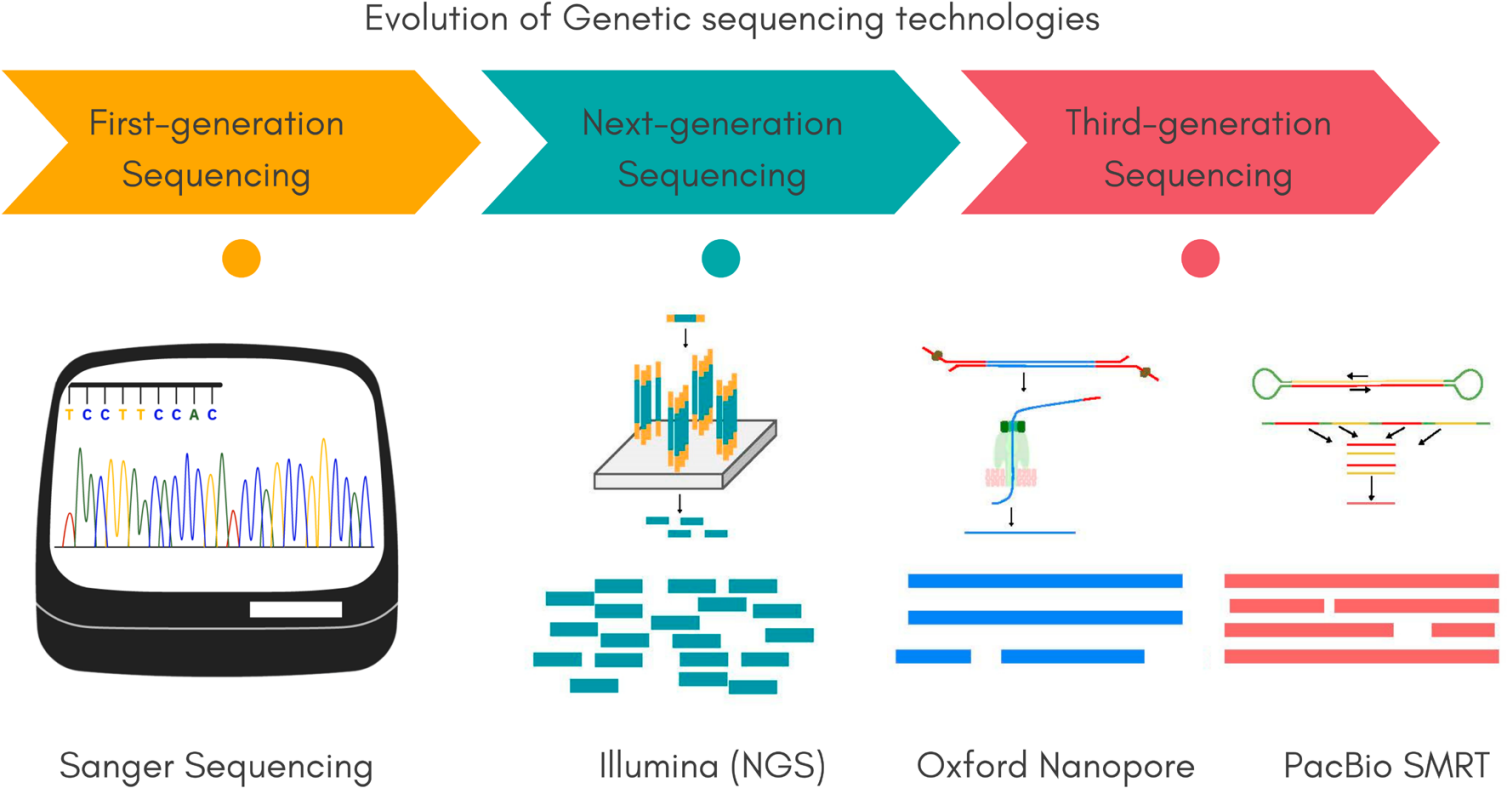
Evolution of Genetic sequencing technologies used in microbial bioprospecting. This figure compares sequencing platforms across technological generations. First‐generation Sanger sequencing offers high basecalling accuracy and is commonly used for phylogenetic marker genes such as 16S rRNA. Second‐generation technologies, such as Illumina, provide high‐throughput short reads ideal for metagenomic profiling, although limited in resolving complex genomic regions. Third‐generation platforms, including Oxford Nanopore and PacBio, generate long reads that span entire operons or genomes, enabling the resolution of structural genomic features and improving assembly quality (Ermini and Driguez 2024).

A number of sequencing technologies are currently available, each with its own distinct advantages and limitations, enabling the generation of increasing volumes of data at decreasing costs (Reuter et al. 2015) (Figure 5). Early molecular ecology studies were based on Sanger sequencing to analyse the 16S rRNA gene—a highly conserved phylogenetic marker—known for its high basecalling accuracy. These studies confirmed the existence of numerous uncultured microbial taxa by comparing environmental 16S rRNA gene sequences with those of known organisms (Foster 2017).

Second‐generation, high throughput platforms such as Illumina, produce massive datasets at relatively low cost but generate shorter reads (150–300 bp), which can hinder genome assembly, particularly in repetitive or highly variable regions (Hu et al. 2021). Despite this limitation, Illumina remains a valuable tool for characterizing complex microbial communities, especially since it bypasses the need for cultivation (Winand et al. 2019) (Figure 5).

In contrast, third‐generation technologies, such as those developed by Pacific Biosciences (PacBio) and Oxford Nanopore Technologies (ONT), produce much longer reads that spans entire operons or chromosomes offering significant advantages for genome assembly and structural variation analysis (Garrido‐Cardenas and Manzano‐Agugliaro 2017) (Figure 5). However, these platforms still exhibit relatively high error rates, requiring greater coverage depth to achieve reliable results and, therefore, raising overall sequencing costs (Lin et al. 2021). Nevertheless, ongoing improvements in platform accuracy and sample preparation methods continue to enhance their utility (Pereira et al. 2020).

High‐throughput sequencing has transformed microbial ecology and taxonomy. Even traditional fields such as species description increasingly depend on genomic data (Hu et al. 2021). In particular, metagenomics provides access to ecosystem‐wide genomic data independent of cultivation (Handelsman 2004; Foster 2017). This approach allows the detection of biosynthetic gene clusters (BGCs) that encode potential antimicrobial compounds, many of which can be further cloned and expressed in laboratory‐friendly host strains (Hua et al. 2015).

On the other hand, metagenomic insights are restricted to available annotated genomes from cultivated organisms. Thus, cultivation remains essential for verifying gene function, validating compound production, and improving genome annotations (Choi et al. 2017; Liu et al. 2022; Ling et al. 2015; Nowrotek et al. 2019). Culturing strategies and metagenomics are currently understood as complementary approaches, with metagenomes offering clues about nutrient dependencies and ecological interactions that guide cultivation efforts (Remenár et al. 2015; Nowrotek et al. 2019).

Given the scale of sequencing data, robust computational tools and reference databases are essential for genome assembly, annotation, and downstream analysis. Bioinformatics pipelines often include gene prediction algorithms like Prodigal and protein domain databases such as Pfam, which are used by platforms like Traitar to infer microbial phenotypes directly from genomic sequences (Weimann et al. 2016). This type of *in silico* phenotyping can inform the design of culture media and isolation strategies.

Recent advances in genome mining and synthetic biology have also enabled targeted searches for new antibiotics. For instance, by screening genomic datasets for signatures of glycopeptide antibiotic biosynthesis, researchers have identified novel compounds that retain vancomycin‐like activity without resistance‐associated elements (Culp et al. 2020; Cook and Wright 2022). These strategies rely on detecting co‐localized resistance and biosynthesis genes within BGCs, an approach that has expanded the discovery of natural product scaffolds.

Beyond discovery, sequencing and bioinformatics have applications in protein modelling, drug design, and the study of antibiotic resistance. These technologies allow for the rapid identification of virulence factors, resistance genes, and mobile genetic elements, providing a deeper understanding of microbial ecology, evolution, and clinical risk (Gwinn et al. 2019; Uddin et al. 2021). As sequencing becomes more affordable and accessible, it is increasingly integrated into laboratory workflows, enriching basic and applied microbiology alike.

Nevertheless, data processing remains a challenge due to the massive volume of information generated. Analysis is influenced by multiple factors, including microbial diversity, sequencing depth, read length, and the choice of assembly and binning algorithms (Meziti et al. 2019, 2021). Despite these complexities, bioinformatics continues to revolutionize drug discovery by guiding the development of more precise data‐driven strategies to combat AMR (Hemlata 2016; Uddin et al. 2021).

## 8 Functional Annotation of Genomes

The sequencing and assembly of genomes have made functional annotation a routine yet essential step in genomic analysis. Approaches for bacterial genome analysis enable the identification of genes, regulatory elements, and biosynthetic pathways, which are central to the discovery of bioactive compounds with pharmaceutical potential. A widely used tool for this purpose is AntiSMASH, which identifies BGCs responsible for the production of secondary metabolites. It allows for the simultaneous analysis of multiple contigs and provides prediction for a variety of compound classes (Mardis 2017; Blin et al. 2021).

For antimicrobial peptide (AMP) discovery, the Antimicrobial Peptide Database (APD) provides curated information and supports sequence‐based identification of AMPs within microbial genomes (Wang et al. 2016). Additional tools include BAGEL, which screens for bacteriocin gene clusters using multiple specialized databases (van Heel et al. 2018), and BACTIBASE, which predicts bacteriocin activity based on calculated physicochemical properties (Hammami et al. 2010). In addition to biosynthetic potential, functional annotation is also critical for AMR gene identification.

Traditional antimicrobial susceptibility testing is often labor‐intensive and may not be feasible for slow‐growing or uncultivable organisms. In contrast, resistance genes can be detected *in silico* by comparing genomic sequences against curated databases. A relevant resource is the Antibiotic Resistance Genes Database (ARDB), which integrates a plethora of publicly available information on resistance genes, mechanisms, resistance profiles, and associated metadata. The database supports sequence similarity searches and includes tools for identifying known resistance mutations (Wade 2002; Liu and Pop 2009; Macesic et al. 2017).

Another important feature frequently annotated in microbial genomes is the CRISPR‐Cas system, a prokaryotic adaptive immune mechanism. These loci consist of short repeated palindromic DNA sequences interspaced with fragments derived from invading genetic elements (i.e., spacers), such as phages or plasmids. This system provides immunity by processing these arrays into small guide RNAs (crRNAs), which direct Cas proteins to recognize and cleave foreign DNA during subsequent infections (Hille and Charpentier 2016). Beyond its natural function, CRISPR‐Cas has become a transformative tool in biotechnology, with applications in genome editing, disease screening, gene therapy and drug discovery (Barrangou and Doudna 2016).

More recently, CRISPR‐based strategies have shown remarkable potential in combatting AMR. Programmable Cas nucleases can be engineered to selectively target and disrupt resistance genes within bacterial genomes, either eliminating the resistant strain or sensitizing it to antibiotics (Gholizadeh et al. 2020). These findings highlight the expanding role of functional genome annotation, not only in characterizing microbial genetic potential but also in harnessing it for therapeutic innovation.

Beyond resistance prediction, bacterial genomics is proving to be an effective tool to improve the efficiency, specificity, and success rate of new antibiotic development. Furthermore, genomic analysis facilitates the identification of a broad array of essential, diverse, and druggable targets throughout the bacterial life cycle. This approach has helped uncover targets without pre‐existing clinical resistance, elucidate modes of action, and identify enzymatic hits that bypass the need for cellular uptake (Chen et al. 2019; Alkatheri et al. 2023).

Whole‐genomic sequencing also allows for the identification of genes essential for bacterial survival, which can be compared across multiple pathogenic species to reveal conserved targets for broad‐spectrum interventions (Przybyla and Gilbert 2022). This promotes rational drug design strategies aimed at inhibiting specific metabolic pathways. In parallel, functional characterisation of proteins with unknown roles can be achieved by integrating genomic data with structural modelling, enabling the mapping of active sites and the prediction of potent enzyme inhibitors (Amoutzias et al. 2022).

Genomic analysis has also contributed to the identification of virulence factors across different bacterial taxa. By mapping genes associated with pathogenesis, researchers can evaluate the efficacy of anti‐virulence drugs and design target‐specific inhibitors to attenuate bacterial fitness without exerting traditional selective pressure (Rentzsch et al. 2020). Finally, the integration of functional annotation with other omics approaches—such as synthetic biology, metagenomics, and metabolomics—has further expanded the toolkit for discovering novel antimicrobial targets and compounds. This convergence of genomic insight with multi‐platform data is accelerating the identification of innovative therapeutic strategies that go beyond conventional antibiotic classes.

## 9 Secondary Metabolites

Microbial secondary metabolites are low molecular weight compounds that typically exhibit potent biological activity and can be identified through the functional annotation of genomes and metagenomes. These compounds, also referred to as natural products, have given rise to numerous molecules with applications in pharmaceuticals, agriculture, and other areas of biotechnology (Bérdy 2012). The availability of secondary metabolic pathway data and the widespread implementation of bacterial genome sequencing have greatly expanded our ability to detect BGCs responsible for the production of secondary metabolites, many of which had not been observed under standard laboratory conditions (Williams et al. 2020).

Two of the most well‐characterized biosynthetic pathways involved in secondary metabolite production are those encoding non‐ribosomal peptide synthase (NRPS) and polyketide synthase (PKS), which are commonly found in BGCs (Amin et al. 2020). NRPS and PKS pathways are responsible for producing a chemically and functionally diverse group of compounds, including (i) the antibiotics erythromycin, penicillin, daptomycin and vancomycin, (ii) the antifungals amphotericin and echinocandin, (iii) the anticancer agents epothilone and bleomycin, (iv) the cholesterol‐lowering agent lovastatin, (v) the immunosuppressants FK506 and cyclosporine, and (vi) the veterinary antibiotics monensin and avermectin (Wang et al. 2011). While the NRPS and PKS systems have been extensively studies, many BGCs remain uncharacterized and may encode pathways for the production of novel bioactive metabolites (Becerril et al. 2018).

In addition, microorganisms with relatively small genomes often produce a different class of natural products knows as ribosomally synthesized and post‐translationally modified peptides (RIPPs) of great interest (Ju et al. 2014). These peptides undergo a variety of post‐translational modifications that enhance their chemical stability, structural diversity and biological activity, improving their efficacy and target specifity. RIPPs represent a rapidly expanding group of natural products with demonstrated antimicrobial potential and are now classified into approximately twenty subtypes, including lanthipeptides, thiopeptides and linear azol(in) e‐containing peptides (LAPs) (Arnison et al. 2013). LAPs, in particular, constitute a large subgroup cytolytic or antibacterial activity, such as streptolysin S, clostridiolysin S and listeriolysin S (Travin et al. 2020).

Despite their notable biosynthetic potential, many secondary metabolite gene clusters remain silent under laboratory conditions or are expressed at very low levels, making their products difficult to detect and characterize (Rutledge and Challis 2015). Furthermore, the chemical space of known natural products often contains redundant scaffolds, and traditional discovery methods are time‐ and resource‐intensive. These challenges have led to the increasing integration of computational approaches to predict, prioritize, and design novel compounds (An et al. 2025). Recent advances in machine learning, structural prediction, and systems biology are accelerating the identification of promising biosynthetic pathways, paving the way for a new era of data‐driven natural product discovery (Prihoda et al. 2021).

## 10 Artificial Intelligence in Antimicrobial Discovery and Resistance Prediction

New approaches are urgently needed to accelerate drug discovery while reducing associated costs and development timelines (Stokes et al. 2020). Artificial Inteligence (AI), particularly machine learning (ML) has emerged as a powerful tool in this effort. These technologies have benefited from the growing availability of large chemical and biological datasets, which enable the development of accurate predictive models. AI facilitates the virtual screening of billions of molecules, target validation, hit prioritisation, and early compound optimization—capabilities that are difficult to achieve using traditional methods (Kiriiri et al. 2020) (Figure 3A).

In the fight against AMR, AI offers multiple strategies, and a key application is the rapid identification of resistance genes, which enables more accurate diagnostics and targeted antimicrobial therapy, ultimately reducing inappropriate prescribing (Lau et al. 2021) (Figure 3B). AI‐powered tools can outperform conventional susceptibility testing by reducing turnaround time and increasing precision (Lingle and Santerre 2019). Databases such as the Comprehensive Antibiotic Resistance Database (CARD) and MegaRES provide the training datasets required to develop models capable of identifying both known and novel resistance genes, as well as predicting minimum inhibitory concentrations (MICs) of multidrug‐resistant bacteria (Alcock et al. 2020; Doster et al. 2020; Nguyen et al. 2019).

ML algorithms are also being applied to predict molecular properties and propose new structural classes of antibiotics. These models enable *in silico* exploration of vast chemical spaces that would be otherwise inaccessible through experimental screening alone (Stokes et al. 2020). One of the most notable examples is the discovery of halicin, a novel broad‐spectrum antibiotic effective against multidrug‐resistant pathogens, identified through a ML model trained to predict *E. coli* growth inhibition (Stokes et al. 2020). Beyond hit identification, AI tools help optimize physicochemical and pharmacokinetic properties, balancing biological activity with desirable drug‐like characteristics (Schneider et al. 2020; Kiriiri et al. 2020). The use of *de novo* molecular design platforms has yielded promising candidates, including abaucin, a narrow‐spectrum antibiotic active against *Acinetobacter baumannii* identified through AI‐driven chemical space screening (Liu et al. 2023).

AI is also transforming the discovery and design of AMPs. ML models can screen protein sequences for antimicrobial activity, generate novel peptides, and predict toxicity. Successful models require well‐balanced training datasets that include both antimicrobial and non‐antimicrobial sequences. Resources such as the Antimicrobial Peptide Database (APD), CAMP R3, and BaAMPs are publicly available for this purpose (Cardoso et al. 2020), though datasets for non‐ AMPs remain limited, leading to potential false negatives (Kurczab et al. 2014). Following data curation, peptide descriptors—such as 2D/3D QSAR parameters and residue patterns—are selected based on the intended application (Rondón‐Villarreal et al. 2014). The resulting model then predicts candidate AMPs, which are validated experimentally for antimicrobial activity and toxicity (Lau et al. 2021). AI models have also been used to design entirely new AMPs with broad‐spectrum activity against multidrug‐resistant bacteria (Pandi et al. 2023).

The integration of AI with metagenomics allows the analysis of large environmental datasets without the need for cultivation (Figure 3A). These tools can extract biosynthetic gene clusters, predict bioactivity, and link metabolomic signals to producer organisms (Pavlopoulos et al. 2023). One example is microbeMASST, a taxonomically informed mass spectrometry search engine that can identify microbial‐derived metabolites and their producers without prior knowledge (Zuffa et al. 2024). Additionally, metabolic modelling is proving useful in the identification of novel drug targets. By simulating metabolic flux and gene knockouts, AI models can highlight essential pathways and propose intervention points (Gwynne et al. 2023).

The integration of AI, machine learning, and omics technologies is reshaping the landscape of antibiotic discovery and microbial biotechnology (Figure 3A,B). These tools not only accelerate screening and prediction but also improve our ability to uncover the hidden diversity of natural products encoded in microbial genomes (Mullowney et al. 2023). By reducing reliance on cultivation and enabling deeper exploration of metagenomic data, AI‐driven strategies offer powerful new pathways to combat emerging microbial threats and address the urgent need for novel antimicrobials.

It is important to recognize, however, that while AI can improve diagnostic accuracy and support more rational clinical decision‐making, the overuse of antibiotics is also strongly influenced by behavioural and social factors—such as patient pressure and misperceptions about antibiotic efficacy (Vazquez‐Lago et al. 2012; Cole 2014). In a recent study, Ito et al. (2025) explored public attitudes toward diagnostic AI systems, focusing on two approaches: (i) the Global‐AI model, which seeks to reduce antimicrobial prescribing by acknowledging the global threat of AMR, and (ii) the Individual‐AI model, which prioritizes individual patient needs without accounting for AMR. The study revealed widespread resistance to full standardisation, with most participants rejecting both standardized protocols and the Global‐AI approach. These findings highlight that ethical concerns and public perception represent significant barriers to the acceptance and effective deployment of AI in antimicrobial stewardship. Therefore, successful AI implementation must be accompanied by educational initiatives and behavioural strategies that address the human factors underlying inappropriate antibiotic use.

Bridging the gap between AI development and effective AMR mitigation involves a range of technical, regulatory, organisational, and human challenges. Systems that leverage integrated data streams, sound governance frameworks, and advanced technological solutions offer a pathway to overcome these obstacles. Expertise bridging the fields of AMR and AI is crucial for the proper design, upkeep, standardisation, and scaling of AI models for infection management, enabling these tools to effectively support physicians in implementing strategies to reduce antimicrobial resistance (Howard et al. 2024).

## 11 Beyond Antibiotics: Novel Approaches to Antimicrobial Therapy

Despite the many challenges and emerging tools for antibiotic discovery, several promising non‐antibiotic strategies are also under investigation. These include naturally derived and synthetic compounds, phage therapy, quorum sensing inhibitors, AMPs, drug repurposing, and novel delivery mechanisms, among others (Brown et al. 2012; Bhardwaj et al. 2013; Stokes et al. 2017; Schwarz et al. 2022; Magana et al. 2020; Boyd et al. 2021; Cook and Wright 2022; Lai et al. 2022; Plotniece et al. 2023; Garcia Jimenez et al. 2023; Shukla et al. 2023). These alternatives offer complementary mechanisms to traditional antibiotics and can help circumvent the escalating issue of AMR.

Bacteriophages—viruses that specifically infect bacteria—are gaining renewed attention as targeted therapies for drug‐resistant infections. Although they were discovered in the early 20th century and rapidly adopted in some regions, early efforts were limited by inconsistent formulation and storage methods (Lin et al. 2017). More recent studies have demonstrated the successful use of phage cocktails to cure chronic infections, positioning phage therapy as a viable alternative to antibiotics (Broncano‐Lavado et al. 2021). One key challenge is the development of phage resistance, similar to antibiotic resistance, driven in part by bacterial CRISPR‐Cas defence systems. To address this, phage‐derived lytic enzymes are being explored as a way to bypass resistance and directly degrade bacterial cell walls (Abdelkader et al. 2019).

However, the narrow host range of bacteriophages and the potential immunogenicity of viral vectors limit their broad clinical application. In recent years, an alternative strategy has emerged: exploiting the natural bacterial conjugation machinery as a delivery system for antibacterial agents. Conjugative plasmids have proven to be efficient vehicles for transferring nucleic acid‐based antimicrobial systems between bacteria. To date, the arsenal of conjugation‐delivered payloads has included both CRISPR‐Cas systems and bacteria‐derived toxins (Derollez et al. 2024).

Recent studies have employed conjugative plasmids, either in cis or in trans, to deliver the CRISPR‐Cas9 system for the targeted elimination of drug‐resistance plasmids (Wang et al. 2019; Rodrigues et al. 2019) or for the direct killing of specific pathogenic strains (Reuter et al. 2021; Ruotsalainen et al. 2019). These approaches have demonstrated high specificity and efficacy both in vitro and in vivo (Song et al. 2022).

In parallel, other engineered systems have focused on the delivery of type II toxin‐antitoxin (TA) modules with diverse mechanisms of action. In these constructs, donor cells express the cognate antitoxin to avoid self‐intoxication, while target bacterial cells are eliminated through a variety of mechanisms. These include chromosomal DNA degradation (e.g., colicin E7), inhibition of DNA gyrase (e.g., CcdB toxin), cleavage of 16S rRNA leading to translational arrest (e.g., colicin E3), or inhibition of cell wall synthesis via phosphorylation of the peptidoglycan precursor UNAG (e.g., Zeta toxin) (Dmowski and Kern‐Zdanowicz 2020; López‐Igual et al. 2019; Shankar et al. 2007; Starčič Erjavec et al. 2015). This novel use of conjugative elements represents a powerful platform for precision antimicrobials, capable of selectively targeting and neutralizing resistant bacterial populations without disturbing the surrounding microbiota.

Another innovative approach is the inhibition of quorum sensing, a bacterial communication system that regulates virulence gene expression. Instead of killing bacteria, quorum sensing inhibitors (QSIs) and quorum quenchers (QQs) disrupt pathogenicity by preventing coordination of group behaviours, such as biofilm formation and toxin production (Bhardwaj et al. 2013). This anti‐virulence strategy reduces selective pressure for resistance and can be used synergistically with antibiotics.

AMPs are short, naturally occurring molecules with broad‐spectrum antibacterial activity. Their advantages include low resistance development, biofilm disruption, and immunomodulatory effects (Magana et al. 2020; Mookherjee et al. 2020). However, clinical application has been limited by toxicity, cost, and susceptibility to protease degradation. For example, polymyxins are effective against Gram‐negative infections but are also nephrotoxic. Novel analogues like MRX‐8 are currently in clinical trials, designed to retain efficacy while reducing toxicity (Lepak et al. 2020). Similarly, bacteriocins, such as colicin‐like peptides produced by *E. coli* and *P. aeruginosa* , represent species‐specific tools for targeting pathogens (Brown et al. 2012).

Additionally, repositioning existing drugs and combining antibiotics with non‐antibiotic agents can yield synergistic effects that reduce resistance risk. For instance, β‐lactamase inhibitors are combined with β‐lactam antibiotics to restore their efficacy, although emerging resistance to these inhibitors remains a challenge (Gatti and Pea 2025). AI‐guided methods are also helping identify promising drug combinations and repurposing candidates (Stokes et al. 2017; Boyd et al. 2021).

Metal‐based antimicrobials—including silver, copper, and gallium—exhibit potent activity against multidrug‐resistant pathogens and are increasingly being explored for clinical and agricultural use (Valente et al. 2021). These compounds may also play a role in oncology, showing promise in treating drug‐resistant cancers. Organometallic chemistry offers a pathway to develop metalloantimicrobials, combining metal elements with functionalized organic ligands to enhance specificity and reduce toxicity (Turner 2024). However, environmental and ecological impacts must be considered in their application and life cycle.

Advances in medicinal chemistry are enabling the rational design of new synthetic antibiotics based on physicochemical modelling and high‐throughput screening (Richter and Hergenrother 2019). One breakthrough strategy involves conjugating antibiotics to siderophores—iron‐chelating molecules that exploit bacterial nutrient uptake pathways—to improve delivery across Gram‐negative cell membranes (Rayner et al. 2023).

On the other hand, the COVID‐19 pandemic underscored the importance of prevention over treatment. Developing vaccines against bacterial pathogens offers a powerful tool to reduce infection incidence and antibiotic usage (Frost et al. 2023). Furthermore, expanding the use of antiviral vaccines, particularly for respiratory viruses that can trigger secondary bacterial infections, may indirectly help curb inappropriate antibiotic use (Lewnard et al. 2020; Jansen et al. 2021).

## 12 Conclusions

The history of antibiotics highlight their deep impact on modern medicine, while also revealing their inherent vulnerability. The misuse and overuse of these drugs, coupled with their release into the environment, have accelerated the emergence and spread of antibiotic resistance among natural bacterial populations. This growing global treat, combined with the stagnation in the development of new antibiotics and the declining involvement of the pharmaceutical industry, remark a deeply concerning outlook.

Bridging the innovation gap will depend on expanding discovery efforts beyond well‐characterized organisms and natural products to explore more complex or overlooked microbial sources. It demands renewed investment in bioprospecting strategies that engage both the public and private sectors, supporting the scientific exploration of new microbial sources. Fortunately, the methodologies for antimicrobial discovery have never been so diverse. With advances in both culture‐dependent and culture‐independent techniques, we are gaining unprecedented access to the untapped metabolic potential of microbial communities, opening new perspectives for identifying bioactive compounds with pharmacological value.

In parallel, the integration of genomics, metagenomics and computational biology—especially AI—has enabled a shift toward more predictive, scalable, and efficient discovery processes. These tools complement traditional strategies and may be key to overcoming the long‐standing bottlenecks in identifying novel classes of antibiotics. While optimizing antibiotic stewardship through appropriate dosing, targeted prescribing, and controlled treatment durations remains a key clinical goal, this strategy may overlook the behavioural and ethical complexities underlying the AMR crisis. Although enhancing diagnostic accuracy—including the incorporation of AI‐based tools—can help reduce inappropriate prescribing, it does not guarantee that clinical decisions will consistently align with the broader public interest, particularly when they conflict with individual patient demands or institutional pressures. To effectively combat AMR and ensure a sustainable pipeline of anti‐infective therapies, a set of integrated, cross‐disciplinary strategies must be pursued, including (i) innovative cultivation strategies to improve access to previously uncultivated microorganisms and uncover novel biosynthetic pathways and metabolites; (ii) antibiotic stewardship to promote the responsible use of antibiotics through optimized dosing, targeted prescription, and controlled duration to minimize resistance selection; (iii) AI and bioinformatics to accelerate the identification of resistance genes and biosynthetic gene clusters, and optimizing drug design and screening workflows; (iv) alternative therapies to advance the development of non‐traditional antimicrobial strategies, such as phage therapy, AMPs, drug repurposing, and antivirulence agents; (v) policy, regulation, and monitoring to implement stringent controls on antibiotic use in healthcare, agriculture, and aquaculture, as well as to strengthen surveillance systems to track resistance patterns and inform evidence‐based policy; and (VI) educational strategies, behaviour change interventions, incentive restructuring, and public ethical debate to align individual preferences and societal benefits.

The future of antimicrobial therapy will depend not on a single solution but on the convergence of technological innovation, ecological awareness, interdisciplinary research, and global cooperation. With sustained effort, it is possible to reverse current trends and usher in a new era of effective, targeted, and sustainable approaches to controlling infectious diseases.

## Author Contributions

The manuscript was prepared by Cunha‐Ferreira and Krüger, with contributions from Peixoto and Vizzotto. Peixoto and Krüger critically revised the manuscript. All authors read and approved the final version.

## Funding

This work was supported by Conselho Nacional de Desenvolvimento Científico e Tecnológico (310565/2021‐9), Coordenação de Aperfeiçoamento de Pessoal de Nível Superior (PROEX‐CAPES ‐ Andreza De Bem), Fundação de Apoio à Pesquisa do Distrito Federal (00193‐00001746/2022). The Faculty of Technology ‐ FT/UnB (SEI 23106.121523/2025‐72).

## Conflicts of Interest

The authors declare no conflicts of interest.

## Data Availability

Data sharing not applicable to this article as no datasets were generated or analysed during the current study.
